# Distance to Healthcare Facility and Lady Health Workers’ Visits Reduce Malnutrition in under Five Children: A Case Study of a Disadvantaged Rural District in Pakistan

**DOI:** 10.3390/ijerph19138200

**Published:** 2022-07-05

**Authors:** Muhammad Shahid, Waqar Ameer, Najma Iqbal Malik, Muhammad Babar Alam, Farooq Ahmed, Madeeha Gohar Qureshi, Huiping Zhao, Juan Yang, Sidra Zia

**Affiliations:** 1 School of Insurance and Economics, University of International Business and Economics (UIBE), Beijing 100029, China; de202159006@uibe.edu.cn (M.S.); h.p.zhao@126.com (H.Z.); 2 Vanke School of Public Health, Tsinghua University, Beijing 100029, China; jam007@uw.edu; 3 Department of Economics, Shandong Business and Technology University, Jinan 250100, China; 202113912@sdtbu.edu.cn; 4 Department of Psychology, University of Sargodha, Sargodha 40100, Pakistan; najma.iqbal@uos.edu.pk; 5 World Health Organization, Peshawar 25000, Pakistan; babaralamm@who.int; 6 Department of Anthropology, Quaid-i-Azam University, Islamabad 44000, Pakistan; 7 Department of Anthropology, University of Washington, Seattle, WA 98195, USA; 8 Department of Economics, Pakistan Institute of Development Economics, Islamabad 44000, Pakistan; madeeha.qureshi@pide.org.pk; 9 Chinese Academy of Science and Technology for Development, Beijing 100029, China

**Keywords:** distance to the healthcare facility, LHW visits, malnutrition, rural Punjab, Pakistan

## Abstract

This study accesses the impact of lady health worker (LHWs) visits in the community and distance to a healthcare facility on the nutritional status of under-five children. Additionally, it explores the perceptions and attitudes of the community about the performance of LHWs. A self-administered instrument was applied to gather data on different parameters, such as children’s height, age, weight, and socioeconomic status from 384 rural households in a marginalized district of Punjab province with the help of a purposive random sampling technique. The binary logistic regression model was employed for the computation of the probability of malnutrition. The prevalences of stunting, underweight children, and wasting in the district were 34.8%, 46.1%, and 15.5%, respectively. The logistic results illustrate that those households in which LHW visits occur regularly within 15 days (OR = 0.28 with 95% CI: 0.09–0.82) have a lower probability of malnutrition prevalence among their children. The distance to the health facility shows that the odds of malnutrition were higher from 3–4 Kilometers (Km) (OR = 2.61, 95% CI: 0.85–8.14), and odds were also higher for the ≥5 km category (OR = 2.88, 95% CI: 0.94–8.82). Children from richer families had lower chances of being malnourished (OR = 0.28, 95% CI: 0.07–1.14). Furthermore, the respondents show a positive attitude towards LHWs. They have given the first rank to their performance being beneficial to mothers and childcare, especially on checkups and safe deliveries, while they have shown negative responses and given lower ranks to their performance due to irregular visits (6th rank) and poor community awareness (7th rank). We conclude that LHWs’ regular visits to targeted households and less distance to healthcare facilities reduce the malnutrition risk in under-five children.

## 1. Introduction

Malnutrition is a serious public health problem. The prevalence of malnutrition is high in many low- and middle-income countries [1]. The occurrence of stunting is highest in the South Asian region, which accounts for approximately 40% of the world’s stunted children. [2]. Malnutrition is above the threshold limit in India, Bangladesh, and Pakistan. [3,4]. Pakistan Demographic and Health Survey 2017–2018 indicates that 38% of children in Pakistan are stunted, 23% are underweight, and 8% are wasted.

The cadre of community health workers in Pakistan is supposed to play a significant role in the referral of children suffering from severe acute malnutrition observed during the growth monitoring of children in their respective areas. In Pakistan, total LHW coverage is less than 50% of the target population, while in the more remote and underprivileged areas, coverage remains extremely low due to the lack of capable community-based females that could be eligible for becoming LHWs [5]. LHWs achieved 90% error-free performance in managing cases of severe acute malnutrition in southern Bangladesh [6], and the cost per child treated through LHW-delivered care (244 USD) was less than the cost per child (259 USD) treated by outpatient-facility-based care [7]. Therefore, having more LHWs and increasing the frequency of their visits could play a vital role in changing the nutritive status of children in their community. If there is less distance to the healthcare facility for SAM referral, then LHWs’ role will be much more important in reducing child malnutrition. Though the distance to health services remains a major challenge for caretakers to join treatment facilities, subsequent severe acute malnourishment treatment reporting rates remain lower than 40% in cases of maximum intervention [8].

Most of the literature on determinants of malnutrition in Pakistan emphasized social, economic, household, individual, and disease factors. There is not sufficient evidence, particularly on the role of LHWs in the growth of children at the local scale. In Pakistan, the available literature only gives a clue that LHWs have an important role in community public health and much emphasis is placed on LHWs’ role in immunization, mother and child health, family planning, etc. However, literature is limited on LHWs’ association with malnutrition in Pakistan. Moreover, previous studies have found a link between distance to health facilities and the population’s general health status, but, there is still a need to dig deeper and generate evidence for policymakers to conduct a study that may specifically determine the relationship between the prevalence of malnutrition and distance to health facilities. 

This current research examined whether LHWs’ visits and distance to healthcare facilities have a significant role in the malnutrition status of under-five children living in rural deprived areas or not. In Punjab, almost every third child under five years of age is stunted, with a majority of stunted children belonging to 11 districts of southern Punjab [9]. Southern Punjab is facing serious threats concerning preschool children’s nutrition indicators. This study was undertaken in Rahim Yar Khan, a socioeconomically deprived district in the southern region of Punjab. In this district, 78.5% of people live in rural areas [10]. The district has one of the highest malnutrition prevalence among 36 districts in Punjab province and the second-highest poverty prevalence in Punjab province [9].

## 2. Materials and Methods

### 2.1. Theoretical Framework

Usually, researchers apply the utility-maximizing model while assessing levels of malnutrition. In doing so, the researchers tend to follow the production function of the household, which presumes that every single child is alive in a division categorized as family [11,12].

N^i^ = n [H, Z, W, C, ε] (1)

where *N^i^* = standard measurement of anthropometry for a child; *C* is used for consumption; *W* is used for a vector of particular children; *H* is used for a vector of particular domestic, socioeconomic, and environmental dynamics; *Z* is used for the vector of health aspects, though *ε* represents a children-specific error term.

The nutrition production function for the reduced specified form for this study is based on:

CIAFi = f (household socioeconomic elements, such as income; environmental factors, such as distance to health facility; community factors, such as LHWs visits, children’s specific factors, such as birth order, age, sex of the child, and εCIAF).

### 2.2. Study Area and Sampling

**Figure 1:**
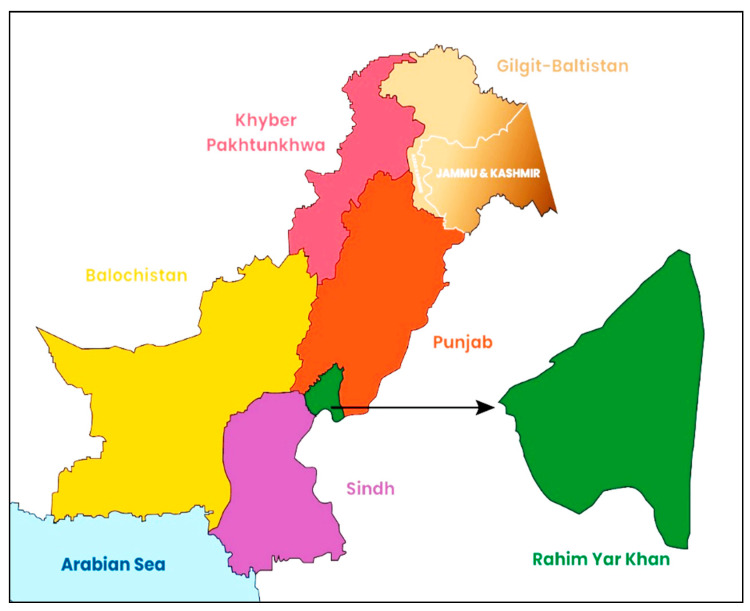
The geographical location of Rahim Yar Khan District on the Pakistani political map.

District Rahim Yar Khan has 128 union councils; out of these, 91 are in rural areas. In district Rahim Yar Khan, 78.5% of people live in rural areas [10], with a 31% literacy rate [9]. Around 93% of the selected households in the district belong to the most deprived category of society. District Health Development Center (DHDC) records show a shortage of LHWs in ~40% of rural UCs and no new hiring of LHWs was opened by the government. In this study, twelve union councils from rural areas of four tehsils were selected for data collection. Out of twelve, five UCs were LHWs. In these five UCs, a vaccinator or a lady health visitor (LHV) visits once a month, and the average distance from governmental basic health facility to most of the population in each rural UC is between 8 and 18 km [DHDC]. In Pakistan, one LHW covers 1000–1500 households in her catchment area [5]. District Rahim Yar Khan is located in the South of Punjab province in Pakistan covering, an area of 11,880 km with two main ecological zones—desert and agricultural ground land. It is divided into four administrative subdistricts/tehsils. The geographical location of the district is highlighted in the Pakistani political map in Figure 1.

The study purposefully included 12 rural UCs from 4 tehsils of Rahim Yar Khan District. Considering a 5% confidence interval with a 95% confidence level, the estimated sample calculated was 384 households. This sample was further allocated to union councils, and the technique applied was “proportionate purposive simple random sampling”.

(2) $$\mathrm{NI}=\frac{n}{N}*\mathrm{Ni}$$

NI = No. of the respondents from each of the union councils; I = no. of union councils in the study area, i.e., 1, 2, and 3; *n =* total sample size (see Table 1).

### 2.3. Data Collection

Using the LHW register record, primary data were collected from 384 randomly selected households through a structured questionnaire. Data were gathered from November 2017 to January 2018. The inclusion of households was based on if under-five children were present in a household. An assessment of 517 under-five children was carried out to take and record anthropometric measurements. The gender-based segregation of these 517 children included 286 (56%) males and 231 (44%) females. Most of the mothers (74%) were illiterate. The study also considered the attitude and experiences of respondents regarding LHWs in the study area. The statements or questions were asked of respondents using the Likert scale (strongly agree to strongly disagree). Informed consent was obtained from all subjects involved in the study through LHWs one week before data collection.

### 2.4. Ethical Considerations

This study was approved in the 6th meeting of the Graduate Research Management Council (GRMC) through the Health Economics Department of the Pakistan Institute of Development Economics (Number: HE-01/2017(PIDE))**.** GRMC works as an institutional review board (IRB). The District Health Officer of Rahim Yar Khan also reviewed and approved survey protocols and tools. Before acquiring the data, all study details were explained to children’s mothers, officers in the health department, and LHWs for their support and cooperation. Around 74% of the mothers in the district were illiterate, and due to cultural and social bonds, they were reluctant to provide written consent. However, all mothers voluntarily participated in the study and agreed to give their oral consent. Mothers were well informed about the study objectives through LHWs in their local language (Saraiki and Punjabi) one week before the survey.

### 2.5. Measuring Important Variables

Dependent variable: Malnutrition was measured through CIAF, as this index is centered on three indexes, i.e., HAZ, WHZ, and WAZ. The cutoff point was set at a standard deviation −2 below the median of WHO standards [13]. According to classification, seven groups of children have formed: (A): no failure; (B): stunted only; (C): wasting only; (D): underweight only; (E): stunted and underweight; (F): wasting and underweight; and (G): stunting, wasting, and underweight. The total amount of child malnourishment occurrence is calculated by combining all groups excluding the A group. A binary variable is used: “1 = child is undernourished” and “0 = child is not undernourished”.

Independent variables: The study measured LHWs’ visits to households in the last 15 days indicator as a binary variable: “1” if LHW visited; otherwise, use “0” if there was no visit in the last 15 days. Another variable distance to the nearest healthcare facility was coded as “1” if the health facility was less than or equal to 2 km, coded “2” if the healthcare center is 3 to 4 km away; and coded “3” if the healthcare center is equal to or greater than 5 km away. The other independent variables used for the research were the gender (such as male, or female), age of the sample (0 to 5 years), and birth order of the sample (1, 2–3, 4–5, and greater than or equal to 6). Household income/wealth status is based on the income of the households (Poor “if the income of the household is less than or equal to 50,000 PKR”; Middle “if family income lies in the range of 50,000 to 100,000 PKR”; and Rich “if family income greater than 100,000 PKR”).

### 2.6. Statistical Analysis

Before testing the relations between malnutrition (CIAF) and (1) LHWs’ visits to households in the last 15 days and (2) distance to the nearest healthcare facility, data were cleaned after the removal of outliers. Z-scores values, falling beyond the World Health Organization’s recommended brackets were removed from the data set. Of the 517 under-five children, the number of children included in the study was 316, and 201 children were spared from analysis because of being outside the range (less than −5 and greater than +5).

The study applied logistic regression for CIAF, which examines the likelihood of child malnutrition. A positive or negative answer was easier to capture; the child being either malnourished or not was coded with the values 0 and 1, respectively. The significance levels were set at *p* < 0.01, *p* < 0.05, and <0.1. The analysis of the study was performed using STATA-15 statistical software.

The binary logistic model’s description is:

CIAFij = Yij = [1 = if the child is undernourished, and 0 = if the child is not undernourished](3)

The reduced-form logistic regression is:

P (Yi = 1|X1i, X2i, …, Xkn) = F (β0 + β1 X1i + β2 X2i + … + βn Xkn)(4)

In the above-mentioned equation, Yi is the dependent variable (CIAF); Xi denotes the explanatory variables; βs represent coefficients, which describes the relationship with the outcome variable CIAF, though Ɛ is the error term.

Furthermore, the study measured the relative importance index (RII) to assess the attitude and experiences of respondents with regard to LHWs’ performance. The RRI index allocates ranks to Likert scale responses, and it assigns a value between zero and one. The 1st rank shows that the respondents’ responses strongly agreed or that respondents have higher satisfaction with that particular statement regarding LHWs’ performance. Similarly, the 7th or lower rank shows the respondents’ bad outcome or that they are highly dissatisfied with that statement or activity of LHWs in the study area. The measurement of the relative importance index is given below:

Relative Importance Index = RII

RII = 5*(n5) + 4*(n4) + 3*(n3) + 2*(n2) + 1*(n1)/A*N(5)

n5 = number of participants who selected strongly agree; n4 = number of participants who selected agree; n3 = number of participants who selected neutral; n2 = number of participants who selected disagree; n1 = number of participants who selected strongly disagree; A = highest weight for Likert scale = 5; N = total number of respondents/households = 384.

## 3. Results

In Rahim Yar Khan District, underweight prevalence rates are 46.1%, whereas stunting and wasting prevalence rates are 34.83% and 15.49%, respectively. Around 83.17% (430) of respondents have above 5 km distance to the nearest healthcare facility, 8.32% (43) of respondents have a 3 to 4 km distance, and 8.51% (44) have a distance less than or equal to 2 km from the nearest healthcare center. Around 75.82% (392) of respondents reported that LHWs have not visited their houses in the last 15 days, while 24.18% (125) reported that they have visited (Table 2).

The logistic regression results are given in Table 3. Those households in which LHWs visited within 15 days (OR = 0.28 with 95% CI: 0.09–0.82) had a lower probability of malnutrition prevalence among their pre-school children. Across the distance to health facility categories, the odds of malnutrition were higher in the 3–4 km category of distance to a healthcare facility (OR = 2.61, 95% CI: 0.85–8.14), and odds were also high for the ≥5 km category (OR = 2.88, 95% CI: 0.94–8.82). Findings showed that the age range of children (age 25–48 months) was correlated with a greater probability of malnourishment. Analysis of birth order and association with malnutrition depicted that likelihood of malnutrition is lower in the case of a higher order of birth, such as children 4–5 years of age (OR = 0.44, 95% CI: 0.21–0.94). Children belonging to rich families had the lowest probability of being undernourished (OR = 0.28, 95% CI: 0.07–1.14).

**Figure 2:**
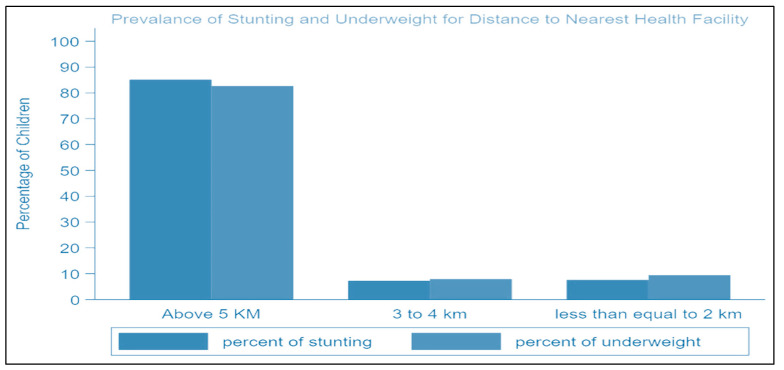
Occurrence rates of stunting and being underweight for distance to the nearest health facility.

Figure 2 highlights that rates of being underweight and stunting increase as the distance to the nearest health facility increases, while wasting is not a major issue in the study area.

**Figure 3:**
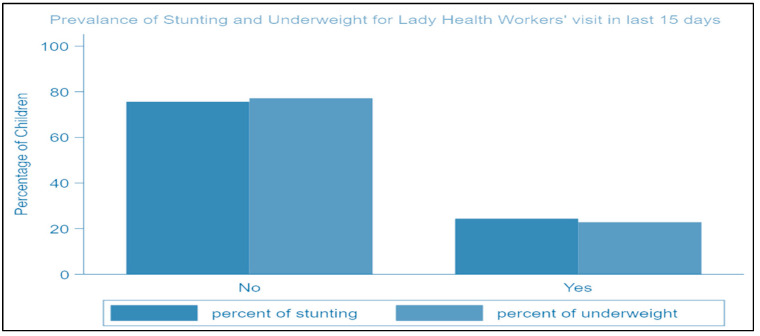
Occurrence rates of stunting and being underweight for LHW visits within 15 days.

Figure 3 highlights that the rates of being underweight and stunting decrease when LHWs have proper visits within 15 days.

Results in Table 4 show that respondents give 1st rank to the statement that LHWs are active in mothers’ and children’s care, especially in females’ pre- and post-checkups and their safe deliveries. The 1st rank shows that the respondents’ responses strongly agreed, or respondents have higher satisfaction with that statement regarding LHWs. Similarly, people gave 6th and 7th ranks to LHW visiting regularly and LHW awareness to the community regarding water, sanitation, and hygiene (WASH), which shows respondents’ bad outcomes or that they are highly dissatisfied with that statement or activity of LHWs in the study area.

**Figure 4:**
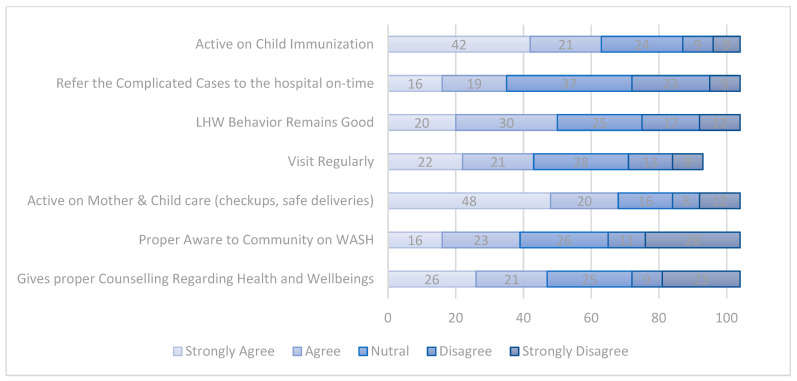
Community opinion on LHWs’ performance.

Figure 4 depicts the same results given in Table 4. It shows the respondents’ responses on the Likert scale regarding their LHWs. The digits in the figure show the number of respondents to that particular Likert response. For example, 42 respondents strongly agreed or were strongly satisfied with LHWs regarding their activeness in child immunization activities in the study area.

## 4. Discussion

This study investigates the influence of LHWs’ visits and distance to healthcare centers on the nutritional status of under-five children in a marginalized rural district of Rahim Yar Khan. Forty percent of the rural areas of the district are without LHWs. Basic health units are far-flung and not easily accessible to the common folk. The logistic results of this study revealed that the probability of child malnutrition was reduced when LHWs ensured and increased regular visits to the targeted population in the study area. The actual coverage of LHWs to the target population is still a serious concern in Pakistan. A study on maternal and child health in Pakistan indicated that there was administrative incompetence and around half of the population in the remote and poorest areas is deprived of LHWs [14]. A study found that long distances to health care facilities along with fewer LHW visits to homes in the last year caused incomplete immunization in Pakistan [15]. A study in the rural Sindh province of Pakistan showed that if women received low-level maternal-child health information from relatives instead of LHWs, they were less likely to give birth at a medical facility [16]. Another study from Sindh Pakistan depicted that LHWs are capable enough to identify SAM children without medical complications, while only 4% of the screened cases received appropriate counseling on medical and nutritional matters [17]. While examining the role of LHWs, particularly on immunization, nutrition, and early childhood illness, a study in two rural districts of Pakistan found that breastfeeding counseling by LHWs was received by 20% of mothers, and the majority of mothers did not know the correct use of ORS and early signs of pneumonia and other childhood diseases [18]. 

A study from South Asia depicted that antenatal and neonatal care service provision and community mobilization activities are significantly linked with a reduction in neonatal mortality and stillbirths [19]. A study in Malawi, Nepal, and Bangladesh, depicted that those women during pregnancy who were visited by an LHW three or more times were more likely to report the use of particular newborn practices [20]. A study found that 43% of newborns in Bangladesh who did not receive LHW visits in the last three days faced feeding or nutritional difficulties [21]. A study from low- and middle-income countries depicted that LHWs’ home visits were associated with improved care-seeking for sick young infants from a healthcare facility [22]. Research in North Ethiopia showed that postnatal home visits by LHWs were low in rural districts, and because of fewer visits, a limited number of women could practice self-care as well as newborn care [23]. As revealed in a study from rural Africa, the likelihood of proper breastfeeding and secure infant growth was higher in the case of a mother who received standard antenatal visits by LHWs [24].

The logistic results of the study show that as the distance to healthcare facilities increases, the risk of child malnutrition also increases. Some studies highlighted that a short distance to a healthcare facility has a significant role in the recovery of children from malnutrition, especially when severe acute malnutrition cases are referred to the basic healthcare facility. A study in southern Ethiopia showed that walking for more than one hour was significantly linked with slower recovery of the child from severe acute malnutrition [25]. Similarly, a study in Northern Ethiopia depicted that a mother traveling less than two hours for basic healthcare facilities has a significant impact on children’s good recovery [26]. A study in Mali found that reducing the average distance from 20–10 km to a basic health facility has a substantial improvement in height-for-age Z-scores [27]. A study in rural western Kenya stated that a 1Km increase in the distance of respondents’ residence from healthcare centers decreases the rate of clinic visits by 34% from the previous distance to seek childcare [28]. A study in Burkina Faso in West Africa established a significant effect of long distance to the health facility on child mortalities and indicated that compared to being near a health facility in a village, the under-five child mortalities were 50% higher where the time to a healthcare center was 4 h [29]. Likewise, a study from Ethiopia depicted that long distance to a health center is a significant determinant of child malnutrition [30]. A collective study in Afghanistan, Chad, Mali, and Niger showed that long-distance to a healthcare facility was one of the major determinants affecting proportional weight gain in young children [31]. A study in Pakistan and Ethiopia depicted that the common barrier to accessing SAM treatment was the long distance to health facilities [32]. A study highlighted that in Southern Punjab District (Multan), Pakistan, most of the population was not satisfied with the services provided in basic health units, and the main reasons behind the bad performance were a shortage of medicine and long-distance to healthcare facilities from residential areas [33]. Another study from Pakistan observed that households living near basic healthcare facilities had lower chances of ARI and diarrhea prevalence among their children and indicated that long distance to basic health centers was significantly associated with increased morbidity prevalence [34,35].

Additionally, the logistic results of the study show that higher order of birth in children 4–5 years of age is associated with decreased probability of malnutrition. Children in the age group 25–48 months have a greater chance of being undernourished in comparison with other age groups. These results are aligned with some previous studies in Pakistan [36,37,38,39]. Additionally, household income decreases the probability of malnutrition in children. There is evidence that amongst the richer and richest families, the prevalence of malnutrition was the lowest [40,41]. Some recent studies from the same region also show that household socioeconomic deprivation (HDS) strongly contributed to child malnutrition, and malnutrition decreased with a decrease in deprivation status [42,43,44,45,46,47]. 

### Limitation of the Study

This study has some limitations. First, it covers only one of the poorest districts of Punjab province in Pakistan, with a limited sample of 384 owing to financial and logistic difficulties. Therefore, the results are for this district only and cannot be generalized to a larger audience. Secondly, we discuss LHW performance in general; however, the main focus remains on the impact of LHWs’ visits to the community and distance to healthcare facilities. This study principally focuses on the demand side (public side), and not the supply side (governmental/system/LHWs), so study results are based on public responses only.

## 5. Conclusions

Results of the study show that the majority of the population was dissatisfied with LHWs’ irregular visits and inability to create awareness. Additionally, lower socioeconomic status in the marginalized region and higher distances to healthcare facilities increased the chances of malnutrition. Therefore, our study necessitates (1) creating community awareness with strong supervision and monitoring of LHWs, (2) equal human development opportunities by allotting due budget for deprived groups in underdeveloped rural areas, (3) providing malnutrition services nearest to communities with regular visits, and (4) providing transportation to ensure regular visits from far-flung rural catchment areas. The results have policy implications for the primary or secondary healthcare department and suggest improvement in the performance of the national LHW Program in Pakistan.

## Figures and Tables

**Table 1:** Sample size allocation from tehsils to union councils.

District	Tehsil	Union Council Name	Availability of LHW	Average Distance to HC Facility	Sample
Rahim Yar	Khanpur	1. Bagh-o-Bahar	Yes	8 km	26
Khan		2. Azeem Shah	No	15 km	34
		3. Kotla Pathan	Yes	12 km	36
	Rahim Yar	4. Bahishti	No	13 km	34
	Khan	5. Sonak	No	17 km	46
		6. Chak No. 84/P	Yes	19 km	35
	Liaquatpur	7. Ghooka	Yes	20 km	25
		8. Shadani	Yes	18 km	26
		9. Trinda Gurgaij	Yes	9 km	30
	Sadiqabad	10. Kot Sanger Khan	Yes	10 km	33
		11. Muhammad Pur	No	15 km	32
		12. Roshan Bhet	No	14 km	27
Total	4	12	Available in UCs = 7		*N* = 384

**Table 2:** Descriptive analysis shows the relationship among various indicators over the dependent variable which is child malnourishment.

Variables	Categories	Frequencies	Percentages	*p*-Values
Gender of Child	Male	93	47.45	0.140
	Female	103	52.55	
Age range of Child (in months)	0 to 12	19	9.69	0.000 ***
	13–24	26	13.27	
	25–36	60	30.61	
	37–48	51	26.02	
	49–60	40	20.41	
Birth Order Number	Birth order 1	52	26.53	0.079 *
	2 or 3	79	40.31	
	4 or 5	41	20.92	
	6 or above	24	12.24	
Income/Wealth Status	Poor	180	91.84	0.008 ***
	Middle	11	5.61	
	Rich	5	2.55	
LHW visit in last 15 days	Yes	47	23.98	0.006 ***
	No	149	76.02	
Distance to Health Facility	≤ 2 km	18	9.18	0.040 **
	3–4 km	18	9.18	
	≥5 km	160	81.63	

Significance level: *** if *p* < 0.01 ** if *p* < 0.05, * if *p* < 0.1.

**Table 3:** Findings of binary logistic regression analysis for CIAF.

Variables	Categories	Odds Ratio	95% CI
Gender of Child	Female (Reference-category)		
	Male	0.79	(0.42, 1. 25)
Age of Child	0–12 months (Reference-category)		
	13–24 months	1.31	(0.55, 3.08)
	25–36 months	2.39 *	(0.85, 6.67)
	37–48 months	7.34 ***	(2.63, 20.52)
	49–60 months	1.04	(0.43, 2.49)
Number of Birth Order	Birth order 1 (Reference-category)		
	2 or 3	0.82	(0.41, 1.67)
	4 or 5	0.44 **	(0.21, 0.94)
	6 or above	0.94	(0.3, 25.57)
Income/Wealth Status	Poor (Reference-category)		
	Middle	3.54	(0.61, 20.54)
	Rich	0.28 *	(0.07, 1.14)
LHW visit in last 15 days	No (Reference-category)		
	Yes	0.28 ***	(0.09, 0.82)
Distance to Health Facility	≤2 Km (Reference-category)		
	3–4 km	2.61 *	(0.85, 8.14)
	≥5 km	2.88 **	(0.94, 8.82)
Significance of the overall model			
Number of observations = 310		Prob > Chi^2^ = 0.0001	
LR Chi^2^ (13) = 54.18		Pseudo R^2^ = 0.1329	
References: Odd ratios and Confidence Intervals			

Significance level: *** if *p* < 0.01 ** if *p* < 0.05, * if *p* < 0.1.

**Table 4:** Community opinion on LHWs’ performance.

Indicators (Statements)	St. Agree (5)	Agree (4)	Neutral (3)	Disagree (2)	St. Disagree (1)	Total (N)	A*N	RII	Rank
1. Gives proper Counselling Regarding Health and Wellbeing	130	84	75	18	23	330	1920	0.17	**4**
2. Proper Aware to Community on WASH	80	92	78	22	28	300	1920	0.16	**7**
3. Active in Mother and Childcare (checkups, safe deliveries)	240	80	48	16	12	396	1920	0.21	**1**
4. Visit Regularly	110	84	84	26	9	313	1920	0.163	**6**
5. LHW Behavior Remains Good	100	120	75	34	12	341	1920	0.18	**3**
6. Refer the Complicated Cases to the hospital on time	80	76	111	46	9	322	1920	0.168	**5**
7. Active on Child Immunization	210	84	72	18	8	392	1920	0.20	**2**

Source: authors’ estimation based on relative importance index (RII).
